# A New Preparation Method for Peroral Endoscopic Myotomy in Patients with Achalasia Can Effectively Reduce the Esophageal Residual Contents: A Comparative Retrospective Study

**DOI:** 10.1155/2022/6953166

**Published:** 2022-02-03

**Authors:** Zehua Zhang, Xiaohan Yan, Bensong Duan, Zhuyun Leng, Haibin Zhang, Jinze Li, Yinghua Zhu, Meidong Xu, Qinwei Xu

**Affiliations:** Endoscopy Center, Department of Gastroenterology, Shanghai East Hospital, School of Medicine, Tongji University, No. 150 Jimo Road, Pudong New District, Shanghai 200120, China

## Abstract

**Methods:**

This retrospective study enrolled 65 achalasia patients who underwent POEM from June 2017 to October 2021. Based on the preoperative diet strategies, patients were divided into carbonated beverage group (*n* = 48) and control group (*n* = 17). Demographic and clinical data, duration of preoperative endoscopy, quality of esophagus cleansing, and patient satisfaction on preoperative procedure were collected and compared. In the current study, we established the quality of esophagus cleansing: Grade A, no remnants or only liquid or frothy discharge; Grade B, a little amount of solid content remained; and Grade C, a large amount of solid content remained.

**Results:**

There were 41 Grade A, 6 Grade B, and 1 Grade C patients in the carbonated beverage group, while there were 8 Grade A, 6 Grade B, and 3 Grade C patients in the control group (*p* value = 0.001). The esophagus cleansing degrees were significantly ameliorated after drinking carbonated beverages in all the three subtypes of achalasia according to the degree of dilatation. The mean duration of preoperative endoscopy was 6.54 ± 2.250 minutes in the carbonated beverage group and 10.27 ± 4.788 minutes in the control group (*p* value = 0.010). The score of patient satisfaction concerning the procedure before the POEM in the carbonated beverage group was 4.5 ± 0.652, while the score in the control group was 4.35 ± 0.702 (*p* value = 0.436). In the multivariate analysis, patient satisfaction was significantly associated with male (odds ratio 0.296, 95% CI: 0.097-0.905, *p* value = 0.033).

**Conclusions:**

Drinking carbonated beverages reduce the duration of preoperative endoscopy and ameliorate the esophagus cleansing degrees without impairing patient satisfaction.

## 1. Introduction

Achalasia is an esophageal motor disorder characterized by a failure of the relaxation of the lower esophageal sphincter (LES) and disturbed esophageal peristalsis, with an estimated prevalence of 0.5–1.0 per 100 000 population per year [1, 2]. The clinical symptoms include dysphagia, regurgitation, chest pain, weight loss, and even pulmonary complications [2].

Current treatment options include peroral endoscopic myotomy (POEM), endoscopic pneumatic dilation, endoscopic botulinum toxin injection, and surgical laparoscopic Heller's myotomy (LHM) [3]. The current treatments are usually effective in alleviating symptoms, with different advantages and drawbacks [4, 5]. These treatments are aimed at eliminating the barrier to the passage of food through reducing the LES pressure and improving the relaxation of LES [6]. POEM, initially described in pig model in 2007 [7], is an endoscopic (scarless) method of myotomy that was first reported in clinical trial in 2010 [8]. POEM enables endoscopists to carry out a myotomy of esophageal circular muscle fibers around the GEJ and into the stomach by a submucosal tunnel. On the basis of multitude clinical trials, POEM has been proved to be effective and safe [6, 9–13], supporting the consideration of it as an initial treatment option for patients with achalasia [14, 15].

After routine fasting for upper endoscopy in achalasia patients, large amounts of retained food frequently remain in the esophagus. Evacuation of the retained contents before anesthesia induction is indispensable in order to prevent regurgitation from flowing into the trachea, even the contamination of the esophageal remnants from flowing into the mediastinum or thoracic or abdominal cavity [16]. To ensure the safety of POEM, preoperative endoscopy with a large channel should be performed to ensure the clearance of the esophageal contents [17]. However, the preoperative endoscopy without anesthesia is always time-consuming and painful, especially for the patients with solid food remained in esophagus. Thus, we aimed to develop an effective and less painful preparation method. We found that drinking large amounts of carbonated beverages in a short time leads to the emesis of the remained esophageal contents due to the high carbon dioxide pressure accumulated in the bottom of the esophagus. Therefore, the major purpose of this study was to compare the efficacy and patients' satisfaction of this newly developed method and previous preparation method in a retrospective design.

## 2. Methods

### 2.1. Study Design

This was a retrospective research conducted in Shanghai East Hospital, a tertiary referral center in China between June 2017 and October 2021. The diagnosis of achalasia is based on Eckardt score, esophagogastroduodenoscopy (EGD), barium esophagography, and high-resolution manometry (HRM). Demographic and clinical data were collected, including patient's age, gender, body mass index (BMI), symptom duration, previous POEM, Eckardt score, Chicago classification (according to high-resolution manometry), degree of esophageal dilatation, and duration of preoperative endoscopy.

A total of 65 achalasia patients who underwent POEM were retrospectively enrolled and were divided into 2 groups according to different preparation methods before POEM. All these patients took proton pump inhibitor (PPI) during around 10 days before POEM. 17 patients took only clear liquid diet (around 3000 ml without carbonated beverage) within 48 hours before the POEM, water (around 2500 ml) within 24 hours before it and fasting within 6 hours before it [18], which was regarded as the control group. 48 patients were required to take only carbonated beverage within 48 hours before the POEM and to fasting within 6 hours before it as well, which was regarded as the carbonated beverage group. These patients were requested to prepare 3 liters of carbonated beverage per day and drink 1 liter at a time in a short period of time to cause emesis to evacuate residual contents.

All the enrolled patients completed a questionnaire concerning the score of satisfactory and tolerance about the preparation before POEM (excellent = 5; very good = 4; good = 3; fair = 2; poor = 1).

Written informed consent was obtained from all patients before enrollment. The study protocol was approved by the institutional ethics committee of Shanghai East Hospital and was performed in accordance with the Declaration of Helsinki.

### 2.2. Definitions

In the Chicago classification system, achalasia consists of three distinct subtypes (types I, II, and III) according to the pattern of esophageal contractility shown by HRM [19]. Dilatation of esophagus is divided into three grades: grade I (diameter of esophageal lumen < 4 cm), grade II (diameter: 4-6 cm), and grade III (diameter > 6 cm or sigmoid type) [20].

**Figure 1:**
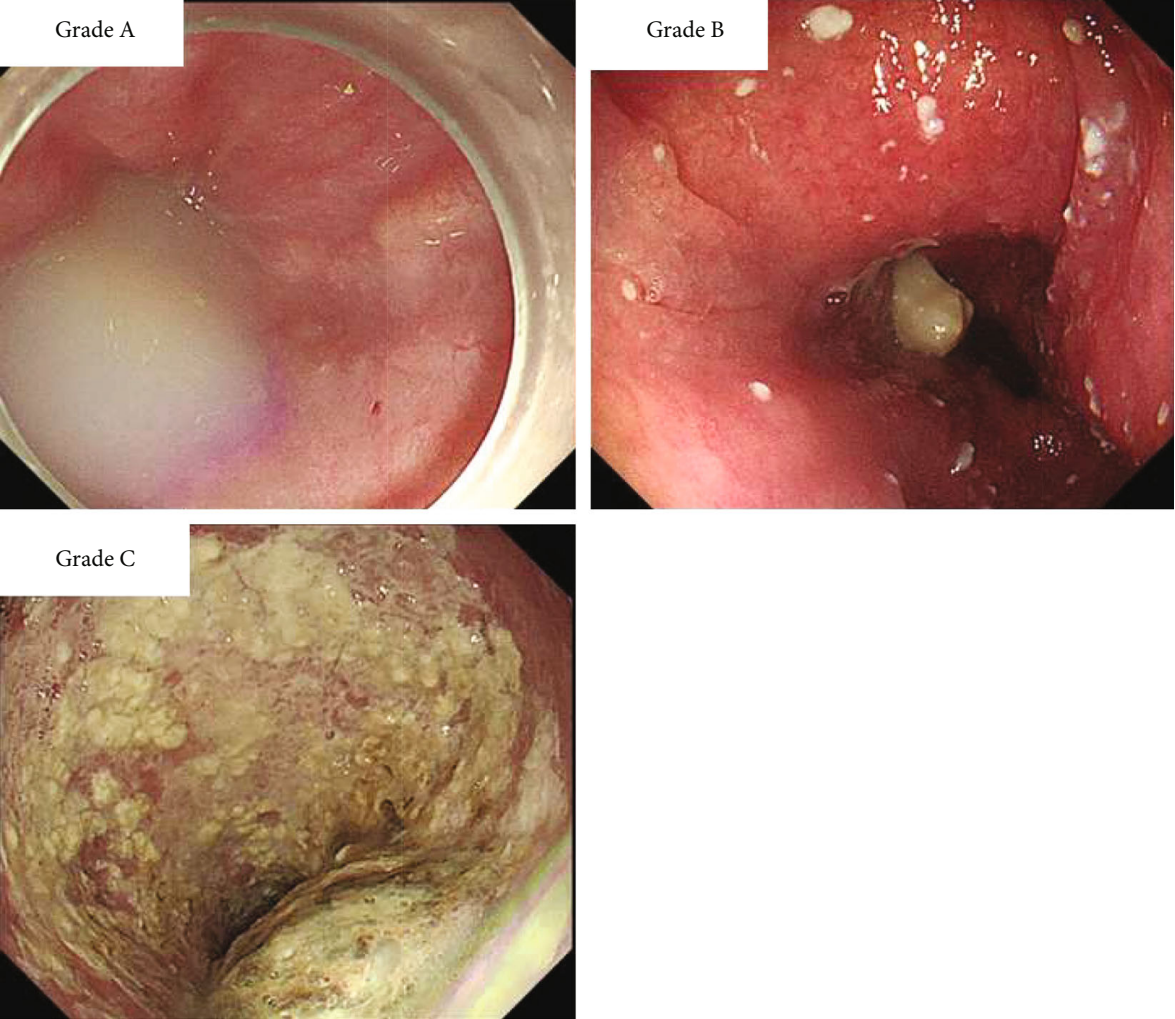
Quality of esophagus cleansing. In the current study, the quality of esophagus cleansing was defined as follows: Grade A (only liquid or frothy discharge or no remnants), Grade B (a little amount of solid food remained), and Grade C (a large amount of solid food remained).

In this study, we defined the quality of esophagus cleansing as Grade A (only liquid or frothy discharge or no remnants), Grade B (a little amount of solid food remained), and Grade C (a large amount of solid food remained), as shown in Figure 1.

### 2.3. Outcomes

The primary outcome was quality of esophagus cleansing. Different subtypes of the two groups were also compared. The second outcome included duration of preoperative endoscopy and patient satisfaction.

### 2.4. Statistical Analysis

Continuous variables were presented as mean ± standard deviation (SD), and their statistical differences were conducted by Student's *t*-test. Categorical variables were presented as percentages and 95% confidence intervals (CIs), and their statistical differences were analyzed with Chi-square test or rank sum test. Predictors of patient satisfaction were assessed by logistic regression analysis. The results were considered statistically significant at a two-sided *p* value of < 0.05. Data were analyzed using commercially available statistical software packages SPSS version 18.0 (SPSS Inc., Chicago, IL, USA).

## 3. Results

### 3.1. Patient Characteristics

A total of 65 consecutive patients were included in this retrospective study. Of these patients, there were 48 patients in the carbonated beverage group and 17 patients in the control group. There were no significant differences between the two groups in terms of age, gender, body mass index (BMI), symptom duration, and previous history of POEM (Table 1). Three patients in the carbonated beverage group and 2 patients in the control group experienced endoscopic pneumatic dilation previously. None of the patients experienced endoscopic botulinum toxin injection previously. All the patients in the carbonated beverage group had vomiting symptoms before POEM, while none of the patients in the control group vomited.

### 3.2. Primary Outcome

There were 41 Grade A, 6 Grade B, and 1 Grade C in the carbonated beverage group, while there were 8 Grade A, 6 Grade B, and 3 Grade C in the control group (*p* value = 0.001) (Table 2). The esophagus cleansing degrees were significantly improved after taking carbonated beverages to cause emesis. In any of the subtypes of achalasia according to the degree of dilatation, the esophagus cleansing degrees were more significantly ameliorated in the carbonated beverage group than the control group (Table 2). In type I and type II of achalasia according to the Chicago classification, the esophagus cleansing degrees in the carbonated beverage group were also significantly ameliorated (Supplementary Table 1). However, in type III of Chicago classification, there were no significant differences between the two groups (*p* value = 0.48) (Supplementary Table 1).

### 3.3. Secondary Outcome

The duration of preoperative endoscopy was significantly reduced in the carbonated beverage group, compared with the control group (6.54 ± 2.250 vs. 10.27 ± 4.788, *p* value = 0.01). There were no significant differences in the patient satisfaction concerning the procedure before the POEM between the carbonated beverage group and the control group (4.5 ± 0.652 vs. 4.35 ± 0.702, *p* value = 0.436). However, female patients showed lower satisfactory score than male ones by monofactor analysis (4.26 ± 0.682 vs. 4.65 ± 0.102, *p* value = 0.017).

In a multivariate analysis, patient satisfaction was significantly associated with male (odds ratio 0.296, 95% CI: 0.097-0.905, *p* value = 0.033), but not with age (*p* value = 0.461), previous history of POEM (*p* value = 0.157), sigmoid type (*p* value = 0.339), or carbonated beverage group (*p* value = 0.405) (Table 3).

### 3.4. Other Findings

In total, there were 27 Grade A, 6 Grade B, and 4 Grade C in type I patients; 17 Grade A, 5 Grade B, and 0 Grade C in type II patients; and 5 Grade A, 1 Grade B, and 0 Grade C in type III patients. There were no significant differences among type I and type II and type III (type I vs. type II, *p* value = 0.571; type II vs. type III, *p* value = 0.753; type I vs. type III, *p* value = 0.535) in quality of esophagus cleansing.

Totally, there were 28 Grade A, 4 Grade B, and 0 Grade C in grade I patients; 19 Grade A, 5 Grade B, and 2 Grade C in grade II patients; and 2 Grade A, 3 Grade B, and 2 Grade C in grade III patients. The quality of esophagus cleansing of grade I patients was better than that of grade III patients (*p* value = 0.0001). The quality of esophagus cleansing of grade II patients was also better than that of grade III patients (*p* value = 0.028). There were no significant differences among grade I and grade II patients (*p* value = 0.142) in quality of esophagus cleansing.

After drinking large amount of carbonated beverages in a short time, none of the patients had upper digestive hemorrhage due to massive vomiting. The incidence of cardiac mucosal laceration syndrome was 0 under the preoperative endoscopy.

## 4. Discussion

In this retrospective study, we compared the preoperative preparation of POEM in achalasia patients with and without drinking large amounts of carbonated beverages in terms of quality of esophagus cleansing, duration of preoperative endoscopy, and patient satisfaction. Our study demonstrates that the quality of esophagus cleansing was better in the carbonated beverage group than the control group. The duration of preoperative endoscopy was significantly shortened in the carbonated beverage group compared with the control group. However, there were no significant differences in patient satisfaction between the two groups.

Unlike in the field of surgery, there have been few studies concerning diet strategies in the field of therapeutic endoscopy. What is more, most strategies are solely based on clinical experience rather than concrete evidence. To the best of our knowledge, this is the first study concerning the preoperative diet preparation of patients with achalasia, and this is also the first study to evacuate esophageal residues by high pressure caused by carbonated beverages. We believe that our results contribute to the guideline revision and determination of the preparation protocol strategy for patients with achalasia.

Massive vomiting may cause cardiac mucosal laceration and hemorrhage. However, the incidence of these complications was 0, as shown in the current study. Therefore, this new preparation method was safe and of high tolerance, which has a great potential to be popularized.

Furthermore, as we all know, it is hard to evacuate the solid retained contents in patients with achalasia even using a large-channel endoscopy. Especially in patients with Grade C of esophagus cleansing quality, it is almost impossible to evacuate all the retained contents. Consequently, the POEM procedure had to be postponed due to the high risk during anesthesia induction, and additional couples of days of fasting are obligated. In the current study, the POEM procedure of 1 patient in the carbonated beverage group and 3 patients in the control group had to be rescheduled because of insufficient evacuation of esophageal residues. A prospective study demonstrated that an early postendoscopic submucosal dissection (ESD) diet protocol resulted in lower healthcare costs, more comfortable nourishment, shorter hospitalization, and higher patient satisfaction, compared with the conventional fasting protocol [21]. Therefore, it is inferred that rescheduling POEM due to poor quality of esophagus cleansing resulting in longer fasting and hospitalization time tends to be associated with lower patient satisfaction. Carbonated beverages which help improving the quality of esophagus cleansing can potentially ameliorate patient satisfaction. It was not shown statistically in the current study owning to the limited cases of Grade C patients. There was no statistical significance in patient satisfaction between the 2 groups probably owning to the limited cases of Grade C patients.

A paradigm change is happening in healthcare. Value-based healthcare is embraced, and patient-centered outcomes are also a growing concern [22]. In the current study, concerning the preparation before POEM, the female patients showed less satisfactory than the male ones. Consistent with previous studies on other digestive diseases, a lower level of satisfaction among female patients was also noted [23]. However, there were no significant differences in patient satisfaction between the carbonated beverage group and the control group. That is to say, vomiting caused by large amounts of carbonated beverages which seemed to bring more pains did not inevitably result in lower patient satisfaction. Furthermore, the duration of preoperative endoscopy decreased significantly in the carbonated beverage group, which may potentially reverse the patient-centered outcomes in pains caused by the vomit.

There were also several limitations in the present study. First, this was a single-center study with a small sample size. Second, this was a retrospective study. Third, a few patients could not tolerate 3 liters of carbonated beverage. The amounts of carbonated beverage taken by each patient were a little bit different. Even with these limitations, this was the first evidence to support the adoption of carbonated beverages as preoperative diet protocol for patient with achalasia. The long-term food stasis and intraesophageal pressure may lead to chronic inflammation of the esophageal mucosa [24]. Additionally, the advantages of preoperative preparation of carbonated beverages in achalasia patients underwent POEM can be easily extended to LHM and other therapeutic endoscopy. Another prospective research with a larger and multicenter study scale should be conducted to further confirm our conclusion. This method might be widely used in clinical practice and adopted in future guideline.

In conclusion, carbonated beverages would help to greatly improve the esophagus cleansing degrees in all subtypes of patients with achalasia according to the degree of dilatation and reduce the duration of preoperative endoscopy. No significant differences were found concerning the patient satisfaction between the carbonated beverage group and the control group.

## Figures and Tables

**Table 1:** Baseline patient characteristics of the carbonated beverage group and the control group.

	Carbonated beverage group (*n* = 48)	Control group (*n* = 17)	*p* value
Age (years)	49.8 ± 17.9	53.5 ± 10.4	0.300
Sex, male	25 (52.1%)	9 (52.9%)	0.951
BMI	19.8 ± 3.1	20.6 ± 3.6	0.214
Symptoms duration (years)	5.8 ± 2.6	6.4 ± 3.1	0.759
Previous POEM	5 (10.4%)	3 (17.6%)	0.436
Eckardt score	8.5 ± 1.8	7.9 ± 1.8	0.238
Sigmoid type	4 (8.3%)	2 (11.8%)	0.648

BMI: body mass index; POEM: peroral endoscopic myotomy.

**Table 2:** Comparison of the quality of esophagus cleansing between carbonated beverage group and control group in the subtypes according to degree of dilatation.

Degree of dilatation	Carbonated beverage group Quality of esophagus cleansing				Control group Quality of esophagus cleansing				*p* value
	Grade A	Grade B	Grade C	Total	Grade A	Grade B	Grade C	Total	
Grade I	23	1	0	24	5	3	0	8	0.015
Grade II	16	2	1	19	3	3	1	7	0.044
Grade III	2	3	0	5	0	0	2	2	0.04
Total	41	6	1	48	8	6	3	17	0.001

**Table 3:** Multivariate analysis of the factors associated with patient satisfaction concerning the preparation before the procedure.

	Odds ratio (95% confidence interval)	*p* value
Male vs. female	0.296 (0.097-0.905)	0.033
Age	1.013 (0.979-1.048)	0.461
Re-POEM	0.272 (0.045-1.651)	0.157
Sigmoid type vs. others	3.287 (0.286-37.773)	0.339
Carbonated beverage group vs. control group	0.591 (0.171-2.039)	0.405

POEM: peroral endoscopic myotomy.

## Data Availability

Data are available on request.

## Supplementary Materials

Supplementary Materials Supplementary Table 1: comparison of the quality of esophagus cleansing between carbonated beverage group and control group in the subtypes according to Chicago classification.
