# Supplementary material for: A New Preparation Method for Peroral Endoscopic Myotomy in Patients with Achalasia Can Effectively Reduce the Esophageal Residual Contents: A Comparative Retrospective Study
Source: Gastroenterol Res Pract. 2022 Feb 3;2022:6953166. doi: 10.1155/2022/6953166 (PMC8831038; doi:10.1155/2022/6953166)
Supplement: Supplementary Materials — Supplementary Table 1: comparison of the quality of esophagus cleansing between carbonated beverage group and control group in the subtypes according to Chicago classification. [file 6953166.f1.docx]

Supplementary Table 1 Compare of the quality of esophagus cleansing between carbonated beverages group and control group in the subtypes according to Chicago classification

|  | Carbonated beverages group  Quality of esophagus cleansing | | | | Control group  Quality of esophagus cleansing | | | |  |
| --- | --- | --- | --- | --- | --- | --- | --- | --- | --- |
| Chicago classification | Grade A | Grade B | Grade C | Total | Grade A | Grade B | Grade C | Total | P value |
| Type I | 23 | 3 | 1 | 27 | 4 | 3 | 3 | 10 | 0.005 |
| Type II | 15 | 2 | 0 | 17 | 2 | 3 | 0 | 5 | 0.027 |
| Type III | 3 | 1 | 0 | 4 | 2 | 0 | 0 | 2 | 0.48 |
| Total | 41 | 6 | 1 | 48 | 8 | 6 | 3 | 17 | 0.001 |
